# Lower Back Pain As the Initial Manifestation of Primary Follicular B-cell Non-Hodgkin Lymphoma of the Bone Marrow: A Case Report

**DOI:** 10.7759/cureus.117438

**Published:** 2026-10-04

**Authors:** Josef Finsterer

**Affiliations:** 1 Neurology, Neurology and Neurophysiology Center, Vienna, AUT

**Keywords:** bone infiltration, chemotherapy, lower back pain, non-hodgkin lymphoma, vertebral fracture

## Abstract

Follicular B-cell non-Hodgkin lymphoma (B-NHL) is a malignant disease of the lymphatic system that primarily affects the lymph nodes. The primary presentation of follicular B-NHL in the bone is extremely rare. The patient, a 53-year-old woman, developed non-radiating lower back pain at the age of 52. Diagnostic evaluation via magnetic resonance imaging (MRI) and positron emission tomography-computed tomography (PET-CT) revealed a solid intraosseous mass in the L3 vertebral body, an L2 compression fracture, and extensive metastatic disease involving the ribs, vertebral bodies, ilium, and cranial vault. Three months after the onset of lower back pain, a CT-guided biopsy of the ilium confirmed the presence of follicular B-NHL lymphoma cells. These cells tested positive for CD10, BCL6, and BCL2, as well as for the minimal residual disease marker t(14;18). Cranial MRI showed osseous lesions in the cranial vault but no intra-axial lesions. No extraosseous metastases were detected. Risk stratification indicated stage IVA, Follicular Lymphoma International Prognostic Index (FLIPI): 4-5, and FLIPI2: 3. Six cycles (one per month) of therapy with rituximab, bendamustine, and intravenous immunoglobulins proved ineffective after five months. Twelve months after symptom onset, the lymphoma progressed and transformed into diffuse large B-cell lymphoma (DLBCL). Various attempts to treat the DLBCL were also unsuccessful. Twenty months after the onset of lower back pain, the patient died of sepsis. In summary, this case demonstrates that follicular B-NHL can initially involve only the bones and manifest solely as lumbar back pain. In summary, this case demonstrates that follicular B-NHL can initially involve only the bones and manifest solely as lumbar back pain. Despite chemotherapy, symptoms may worsen, and B-NHL may transform into fatal DLBCL.

## Introduction

Primary bone lymphomas (PBLs) are rare extranodal non-Hodgkin lymphomas (NHLs) that originate directly within the bone tissues or bone marrow without initial distant visceral or widespread nodal involvement [1]. PBLs account for about 5% of all primary bone tumors. The most common histological subtype of PBL is diffuse large B-cell lymphoma (DLBCL), which accounts for 80% of PBL cases. Rare PBL subtypes include marginal zone lymphoma, anaplastic large cell lymphoma, T-cell lymphomas, Hodgkin lymphoma, and follicular lymphoma [1]. Follicular B-NHL accounts for less than 1% of all PBL [2,3].

Follicular B-cell NHL (B-NHL) is a malignant disease of the lymphatic system that originates primarily from lymph node follicles [1,2]. Follicular B-NHL is characterized by diffuse lymphadenopathy, splenomegaly, and rarely secondary bone marrow involvement. Secondary infiltration of the bone marrow by a primary nodal follicular B-NHL must be differentiated from primary bone follicular B-NHL. Clinically, follicular lymphoma manifests with fever, night sweats, and weight loss. Diagnosis relies on the histology of a biopsy taken from a lymph node or other affected tissue. Immunohistochemical staining is positive in virtually all cases for the cell surface markers CD19, CD20, and CD10, as well as for monoclonal immunoglobulins; cytoplasmic expression of the BCL-2 protein is also observed. The majority of cases exhibit the characteristic t(14;18) translocation involving the IgH and BCL-2 genes [1]. Risk stratification utilizes the Follicular Lymphoma International Prognostic Index (FLIPI), which considers five independent predictors of poorer survival: age >60 years, hemoglobin <12 g/dL, elevated serum lactate dehydrogenase (LDH), Ann Arbor stage III/IV, and involvement of more than four lymph node regions. The presence of 0-1, 2, or ≥3 risk factors defines low-, intermediate-, or high-risk disease, respectively [1,2].

Primary bone follicular B-NHL has been described in only a few cases to date [4-7]. To the best of our knowledge, no case of primary bone follicular B-NHL presenting with lower back pain as the initial symptom has been reported. We report here on a woman patient with primary bone follicular B-NHL, initially manifesting solely as localized lumbar pain without radiation. This case is of interest to neurologists and oncologists because the initial symptom of the disease is one of the most common neurological symptoms, and because a malignancy can manifest with such a common and unspectacular symptom.

## Case presentation

The patient is a 53-year-old woman, height 152 cm, weight 58 kg, with a medical history of migraine, cataract surgery, hypothyroidism, and repeated curettages, who was diagnosed with primary osseous follicular B-NHL stage IVA, FLIPI: 4-5, FLIPI-2: 3, at the age of 52 years after having developed lumbar pain without radiation three months earlier. The back pain was described as undulating, up to a VAS score of 6, was not dependent on position or activity, and did not disrupt sleep. She also reported increased sweating following the onset of pain, but there were no concomitant symptoms such as fever, weight loss, fatigue, or anorexia. The patient had no history of malignancy.

Work-up for lower back pain by magnetic resonance imaging (MRI) revealed a solid intraosseous mass lesion in L3 and an impression fracture of L2, but no disc prolapse, vertebral stenosis, cord infarction, myelopathy, or infiltration of the spinal cord (Figure 1). Positron emission tomography-computed tomography (PET-CT) showed multiple foci of hypermetabolism in the spine, proximal portions of the long bones, and right os ileum, a pathological fracture of L2, but no extraosseous lesions (Figure 2). A CT-directed bone biopsy of the right os ileum revealed B-NHL, which was CD10+, BCL6+, and BCL2+. In contrast, cytological processing of the bone marrow biopsy yielded no evaluable results. Histological examination of the bone marrow biopsy was not representative of B-NHL, and flow cytometry showed no evidence of infiltration by B-NHL. However, minimal residual disease (MRD) marker Kiel (t(14;18)) was detectable. These results show that targeted puncture of lesions is more effective than an untargeted bone marrow biopsy. Cerebral MRI showed osseous lesions in the cranial vault, but no intra-axial lesion. Six cycles of rituximab (375 mg/m^2^) and bendamustine (120 mg/m^2^) plus intravenous immunoglobulins (IVIGs) every four weeks were ineffective.

**Figure 1:**
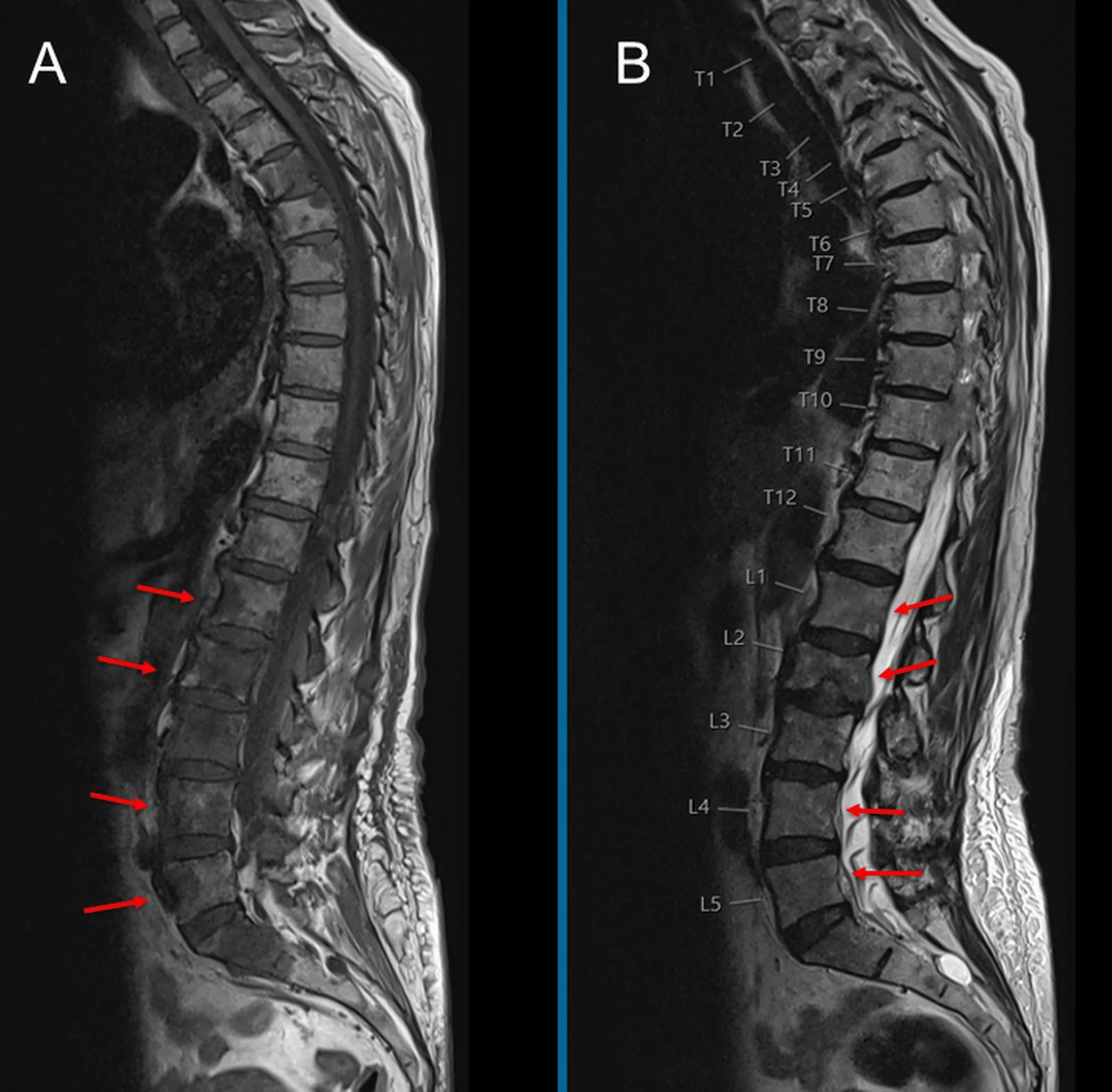
MRI of the lumbar spine, T1-weighted imaging (panel A) and T2-weighted imaging (panel B), showing numerous solid intraosseous focal lesions (arrows). A solid focal lesion in the third lumbar vertebra (L3) breaches the dorsal cortex on the right side and infiltrates the epidural space.

**Figure 2:**
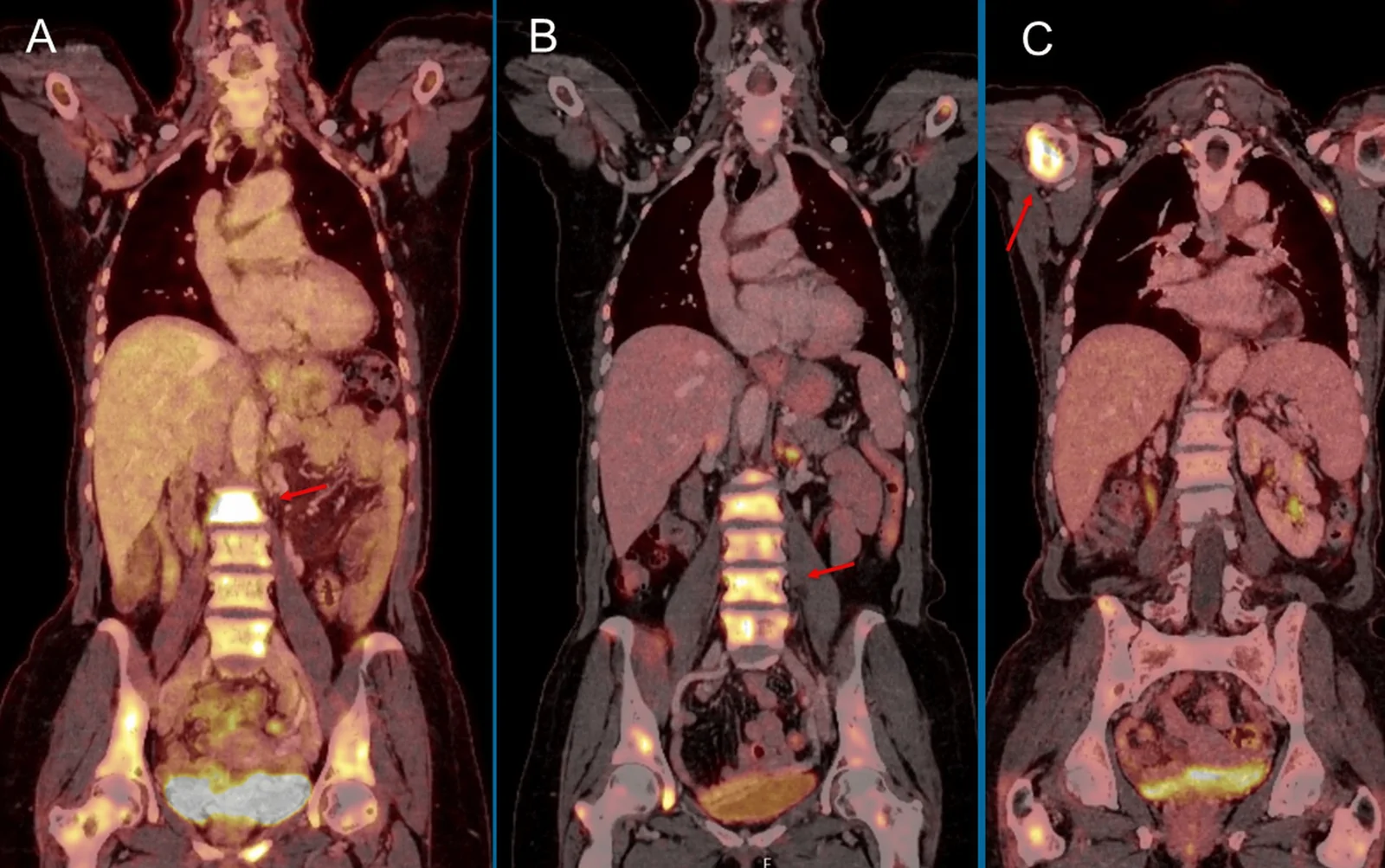
Whole-body PET-CT from October 2024 (panel A), July 2025 (panel B), and November 2025 (panel C) showing multiple foci of tracer uptake throughout the axial skeleton and in the proximal sections of the long bones (arrows). There is a pathological vertebral body fracture (collapse) at the L2 level. PET-CT: positron emission tomography-computed tomography.

Ten months after the B-NHL diagnosis, symptoms such as lumbar pain and sweating worsened. Clinical neurological examination revealed dysgeusia (present since the start of valaciclovir therapy), painful neck muscles, a positive snout reflex, and diminished Achilles tendon reflexes. Current medication consisted of L-thyroxine, valaciclovir, zolmitriptan, vitamin D, pantoprazole, and filgrastim. MRI imaging showed an increase in lesion burden, with foci in the cranial vault as well as the thoracic and lumbar spine, and a soft-tissue component at levels T9-11 and L2-3 (Figure 3). PET-CT revealed a pathological fracture of the left second rib and partial regression of previously described lesions, but also new foci in the left ilium, left acetabulum, sacrum, and vertebral bodies T9 and T10 (Figure 2). A second bone marrow biopsy showed transformation of the B-NHL into DLBCL (not otherwise specified (NOS)), germinal center B-cell-like (GCB) subtype, with a BCL2/c-MYC protein expression score (green) of 1. Treatment with polatuzumab vedotin plus rituximab, gemcitabine, and oxaliplatin, as well as radiotherapy, proved ineffective (Figure 3). Consequently, therapy was switched one month later to glofitamab, gemcitabine, and oxaliplatin. A PET-CT scan performed three months later showed a mixed response to this treatment (Table 1). As the DLBCL continued to progress, treatment with ifosfamide, carboplatin, and etoposide was initiated two months later (Figure 3). Unfortunately, the patient passed away two months later.

**Figure 3:**
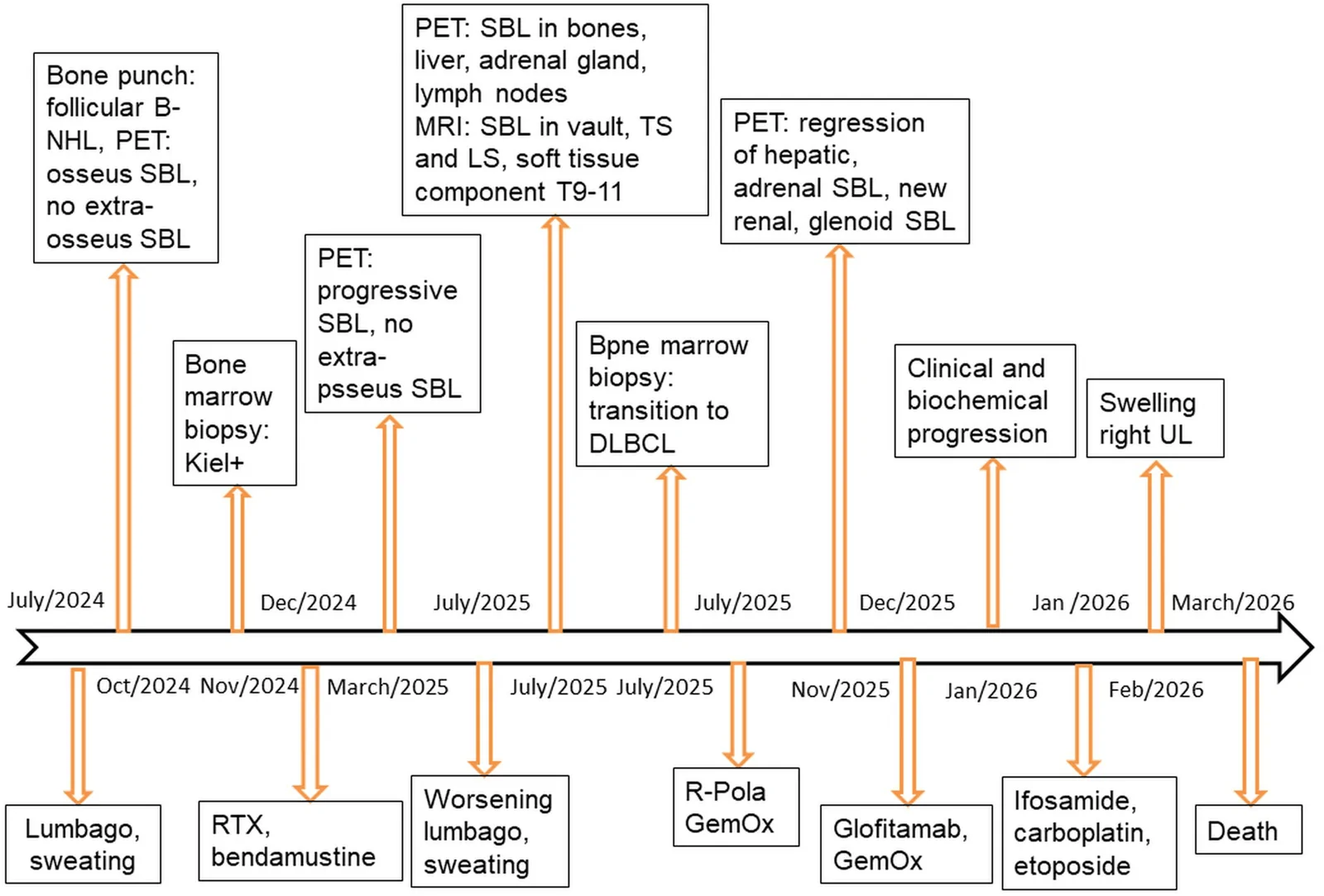
Course of the disease over time. Feb: February, Dec: December, Jan: January, LS: lumbar spine, Nov: November, Oct: October, Pola-R-Gemox: polatuzumab, rituximab, gemcitabine, oxaliplatin, RTX: rituximab, SBL: secondary blastoma, TS: thoracic spine, UL: upper limb, B-NHL: B-cell non-Hodgkin lymphoma, MRI: magnetic resonance imaging, DLBCL: diffuse large B-cell lymphoma, PET: positron emission tomography.

**Table 1:** Findings from four PET-CT scans performed during the course of the disease. Nm: not mentioned in the report. PET-CT: positron emission tomography-computed tomography.

Date of examination/abnormalities	October 2024	March 2025	July 2025	November 2025
Liver hemangioma (segment VII)	Yes	Yes	Yes	Yes
Multiple focal tracer uptakes (lytic and blastic) in the axial skeleton	Yes	↓	↑ (+ soft-tissue component)	↓
Multiple focal tracer uptakes (lytic and blastic) in the long bones	Yes	↓	↑	↓
Os ileum	Yes	↓↑	Nm	↓
Acetabulum	No	Yes	Nm	Nm
Sacrum	No	Yes	Yes	Nm
Pathological fracture L2	Yes	Yes	Yes	Nm
Pathological fracture second rib	No	Yes	Consolidated	Nm
Liver	No	No	Yes	No
Adrenal gland	No	No	Yes	No
Glenoid	No	No	No	Yes
Kidney	No	No	No	Yes
Axillary lymph nodes	No	No	No	Yes

## Discussion

The index patient is of interest due to the presence of primary osseous follicular B-NHL and the fact that symptoms were only mild at the onset of the disease. Previously described patients with primary osseous follicular B-NHL exhibited local bone pain (including at rest), swelling or palpable masses in the affected bone (particularly the pelvis, spine, and long bones), pathological fractures resulting from no or minimal trauma, and pain-induced limitations of movement (especially with joint involvement) [3-7]. Furthermore, primary bone lymphoma rarely metastasizes to parenchymal or hollow organs [7]. Bones in which primary bone follicular lymphoma has been described include the mandible [5], palate [8], skull (index patient), pelvis (index patient), ribs (index patient), leg bones, humerus, and vertebral bodies (index patient). Regardless of the location, primary bone lymphoma is best detected via MRI and PET-CT [9]. To distinguish the most common causes of lower back pain from primary bone lymphoma, it is necessary to perform an MRI of the lumbar spine and, if lymphoma is suspected, to follow up with a fluoro-deoxy-glucose (FDG)-PET scan. Differential diagnoses of B-NHL that should be considered include osteosarcoma, Ewing sarcoma, chondrosarcoma, chronic osteomyelitis, Langerhans histiocytosis, multiple myeloma, metastatic malignancy, bony tuberculosis, brucellosis, and other primary bone lymphomas. These differential diagnoses can best be distinguished from B-NHL by means of a targeted biopsy, appropriate bacterial culture, and immunohistochemical analysis.

Another important aspect is that the initial bone marrow findings were unremarkable, which led to the diagnosis being overlooked. Therefore, when primary bone lymphoma is suspected, MRI and PET-CT scans should be performed in addition to bone marrow aspiration. Once a relevant lesion is identified, a CT-guided biopsy should be carried out to confirm the diagnosis and avoid delaying the start of chemotherapy. If treatments involving bendamustine, IVIGs, and rituximab prove ineffective, the rituximab, cyclophosphamide, doxorubicin, vincristine, and prednisone regimen (R-CHOP) may be attempted [10]. However, as the present case demonstrates, other therapeutic approaches--including local radiotherapy--can also yield favorable results.

The third point concerns the transformation of follicular B-NHL into DLBCL after ten months. The transformation rate from follicular B-NHL to DLBCL is estimated at 2-3% per year [11]. The estimated cumulative lifetime risk of such a transformation is 20-30% [11]. Approximately 90% of all histological transformations originating from follicular B-NHL result in DLBCL [11]. The risk of transformation depends on various factors: the B-NHL subtype, the time course (the highest risk exists in the first few years following diagnosis), the presence of mutations in the TP53, MYC, BCL2, and BCL6 genes, acquired immunodeficiencies (e.g., due to HIV, transplantation, rheumatoid arthritis, Sjögren's syndrome, or lupus erythematosus), exposure to toxins (benzene, herbicides, pesticides, trichloroethylene), and the type of prior treatment (rituximab-based therapy can reduce the risk of transformation) [12]. The survival times of the previously described patients with primary follicular B-cell NHL of the bones [4-7] were similar to those of the index case.

## Conclusions

In summary, this case demonstrates that persistent, atypical, or red-flag lower back pain warrants appropriate imaging and consideration of malignant causes. In cases of unexplained lower back pain with atypical features (e.g. sweating), MRI of the lumbar spine and PET-CT should be considered. Chemotherapy with rituximab, bendamustine, and IVIGs can be of limited benefit in single cases of primary bone follicular B-NHL and primary bone follicular B-NHL can lead to transformation into DLBCL. This case expands the spectrum of clinical presentations of B-NHL, as the primary bone follicular B-NHL manifested clinically exclusively as lower back pain. Clinicians should consider the possibility that lower back pain may be caused by an intraosseous lymphoma. Future research should focus on the development of more effective diagnostic methods and treatments for follicular B-NHL.
